# Electrospun Polyvinyl Alcohol/Sodium Alginate Nanocomposite Dressings Loaded with ZnO and Bioglass: Characterization, Antibacterial Activity, and Cytocompatibility

**DOI:** 10.3390/polym17162185

**Published:** 2025-08-09

**Authors:** J. Andrés Ortiz, Francesca Antonella Sepúlveda, Siomara Flores, Marcela Saavedra, Suhelen Sáez-Silva, Thomas Jiménez, Paola Murgas, Scarlett Troncoso, Camila Sanhueza, María T. Ulloa, Lorena Porte Torre, Manuel Ahumada, Teresa Corrales, Humberto Palza, Paula A. Zapata

**Affiliations:** 1 Laboratorio Química de Biomateriales, Departamento de Ciencias del Ambiente, Facultad de Química y Biología, Universidad de Santiago de Chile (USACH), Santiago 9170022, Chile; 2 Departamento de Ingeniería Química, Biotecnología y Materiales, Facultad de Ciencias Físicas y Matemáticas, Universidad de Chile, Avenida Beaucheff 851, Santiago 8370456, Chile; 3 Grupo Polímeros, Departamento de Ciencias del Ambiente, Facultad de Química y Biología, Universidad de Santiago de Chile (USACH), Santiago 9170022, Chile; 4 Escuela de Medicina, Facultad de Medicina, Universidad San Sebastián, Sede de la Patagonia, Lago Panguipulli 1390, Puerto Montt 5501842, Chile; 5 Laboratorio Centro I+D+i Vertebral-COANIQUEM, Santiago 9020070, Chile; 6 Laboratorio Clínico, Clínica Alemana, Facultad de Medicina Clínica Alemana, Universidad del Desarrollo, Santiago 7610658, Chile; 7 Escuela de Biotecnología, Facultad de Ciencias, Ingeniería y Tecnología, Universidad Mayor, Camino La Pirámide 5750, Huechuraba 8580745, Chile; 8 Centro de Nanotecnología Aplicada, Facultad de Ciencias, Ingeniería y Tecnología, Universidad Mayor, Camino La Pirámide 5750, Huechuraba 8580745, Chile; 9 Grupo de Fotoquímica, Departamento de Química Macromolecular Aplicada, Instituto de Ciencia y Tecnología de Polímeros, C.S.I.C., Juan de la Cierva 3, 28006 Madrid, Spain; 10 IMPACT, Center of Interventional Medicine for Precision and Advanced Cellular Therapy, Santiago 7590000, Chile

**Keywords:** polyvinyl alcohol, sodium alginate, wound dressing, ZnO, bioglass, cytotoxicity, antibacterial activity

## Abstract

Chronic wounds pose a great challenge due to their slow healing and susceptibility to infections, hence the need for innovative alternatives to conventional antibiotics, as increasing bacterial resistance limits the efficacy of current treatments. This paper addresses the development of novel electrospun membranes based on polyvinyl alcohol (PVA) and sodium alginate, incorporating therapeutic ZnO and bioglass (54SiO_2_:40CaO:6P_2_O_5_) nanoparticles. While nanocomposites presented smaller fiber diameters than pure polymers, ternary nanocomposites displayed higher values, e.g., in porous areas, values were in the ca. 80–240 nm range and 0.06–0.60 μm^2^, respectively. The Young’s modulus of the PVA/SA membrane, initially 15.9 ± 2.0 MPa, decreased by 65% with 10 wt.% ZnO NPs, whereas 10 wt.% BG NPs increased it by 100%. The membranes demonstrated efficacy against Gram-positive bacteria, including methicillin-resistant *Staphylococcus aureus* (MRSA) isolated from a human wound secretion, as well as two ATCC strains: *Staphylococcus aureus* and *Staphylococcus epidermidis*. A cell viability assay conducted with HaCaT cells demonstrated nearly complete survival following 72 h of membrane exposure. Their combined Gram-positive antibacterial activity and cytocompatibility support their potential application as biofunctional dressings for the management of chronic and hospital-acquired topical infections, while also contributing to the global effort to combat antibiotic resistance.

## 1. Introduction

The skin plays a vital role as a protective barrier, defending the body from external threats, regulating temperature, and preventing water loss. When damaged by external factors or compromised by immune system dysfunction, the skin may develop wounds that can be either acute or chronic. To repair them, the body initiates the complex wound-healing process, which is frequently hindered by pathogenic bacterial attacks [1]. Medical treatment is often necessary to complement the body’s natural healing process, increase recovery times, and reduce patient discomfort, especially in the case of chronic wounds that require extended and specialized treatment. The global wound care market is expected to grow from USD 19.8 billion in 2021 to USD 27.8 billion by 2026, propelled by an increased susceptibility to skin injuries from burns, surgery, and trauma [2]. In addressing this global issue, electrospun nanofibrous membranes have gained considerable attention, as wound dressing enhances the natural wound-healing process. Their high porosity, large surface area, and suitable mechanical strength, combined with a nano-network structure that closely mimics the skin’s natural extracellular matrix (ECM), make electrospun nanofibrous membranes highly promising for wound treatment [3].

Furthermore, these nanofiber membranes can act as carriers for the controlled delivery and release of biomolecules, drugs, and nanoparticles, expanding their application in wound healing therapies [4]. In this context, polyvinyl alcohol (PVA) has been widely used in the fabrication of electrospun membranes, especially in biomedical applications, due to its biocompatibility, biodegradability, non-toxic nature, and hydrophilicity. PVA is known for its excellent fluid absorption capacity and low protein adhesion, making it ideal for tissue regeneration and wound healing [2,5]. Additionally, PVA can be combined with biopolymers to enhance their functionality and bioactivity [6]. Specifically, PVA-based membranes incorporating sodium alginate (SA) have been developed because the distinctive properties of SA—such as high biocompatibility, low immunogenicity, cost-effectiveness, remarkable gel-forming ability, and structural similarity to proteoglycans (glycosaminoglycans, GAGs)—are essential components of the ECM [7,8].

Combining these properties of PVA with the advantages of therapeutic nanoparticles (NPs) is another promising approach to enhancing the functionality of wound dressings [9]. Bioglass (BG) NPs are inorganic materials composed of SiO_2_-CaO-P_2_O_5_, widely recognized for their bioactivity, especially in bone regeneration, which enhances hydroxyapatite formation and stimulates osteogenic cells. These NPs release beneficial ions such as Na, Ca, Si, and P, activating and regulating osteogenic genes when dissolved in the body’s natural physiological environment [10]. They have also proven effective in wound healing by promoting angiogenesis, increasing vascular endothelial growth factor (VEGF) expression, and enhancing blood coagulation [11]; therefore, BG NPs have garnered interest in the development of biofunctional wound dressings [12,13].

ZnO is another recognized therapeutic NP that has been incorporated into various materials for wound treatments, mainly due to its antibacterial properties [14,15] and safety classification (GRAS) according to the US Food and Drug Administration (FDA) [16]. Compared to bulk zinc oxide, ZnO NPs possess unique chemical, mechanical, and structural properties, which allow for better interaction with cellular biomolecules and easier cellular uptake [17,18,19]. Research has shown that, aside from their highly antibacterial behavior, ZnO NPs are biocompatible, self-cleaning, skin-friendly, and suitable for various biomedical applications, including sunscreens [20] and electrospun membranes with wound-healing potential [21,22,23].

Despite the advantages of BG and ZnO NPs for the design of functional wound dressings, only a few studies have explored the synergistic effect of both particles simultaneously incorporated into polymer matrices. Together, both particles led to improvements in bioactivity, cell viability, mechanical properties, and antibacterial activity compared to membranes containing only a single type of nanoparticle [24,25]. For example, Canales et al. demonstrated that adding BG NPs to PLA/ZnO nanofibers can reduce cytotoxicity while achieving high antibacterial activity, which is attributed to ZnO NPs [24]. The present study aimed to design novel electrospun PVA/SA-based membranes loaded with therapeutic ZnO and BG NPs, either alone (composites) or mixed (ternary composites), for potential use as antibacterial wound dressing prototypes. To our understanding, no studies have reported on PVA/SA/ZnO/BG multifunctional nanofibers. Our PVA/SA/NPs nanofibers were characterized in terms of their morphological, thermal, and mechanical properties to assess their suitability as scaffolds. Additionally, the in vitro antibacterial activity against clinically relevant Gram-positive and Gram-negative bacteria, including *methicillin-resistant S. aureus* (MRSA), involved in skin infections, as well as the cytotoxicity with human keratinocyte cells, was evaluated to provide insights into the potential of these prototypes as antibacterial wound dressing candidates.

## 2. Materials and Methods

### 2.1. Materials

The reagents used in the synthesis of the ternary bioglass (BG) nanoparticles (NPs) were calcium nitrate tetrahydrate (Ca(NO_3_)_2_∙4H_2_O, ACS reagent grade, 99%, Merck), tetraethyl orthosilicate (TEOS, Si(OC_2_H_5_)_4_), reagent grade, 98%, Sigma-Aldrich), ethanol (99.85%, EQUILAB), citric acid (ACS reagent grade, 99.5%, Sigma-Aldrich), ammonium dihydrogen phosphate (NH_4_H_2_PO_4_, 99% Merck), aqueous ammonia (NH_4_OH, ACS reagent grade, 28% *w*/*v*, Merck) and polypropylene glycol (PPG, average molecular weight (Mw) 600 g/mol, Sigma-Aldrich). For the synthesis of zinc oxide (ZnO) NPs, the reagents used were zinc chloride (ZnCl_2_, reagent grade, 98%, Sigma-Aldrich), isopropyl alcohol (98%, Sigma Aldrich), and sodium hydroxide pellets for analysis (Merck). For the electrospun fibers, poly(vinyl alcohol) (PVA) with an Mw of 85,000–124,000 g/mol, sodium alginate (SA) of medium viscosity with an Mw of 216,000 g/mol, a M/G ratio determined by ^1^H NMR of 1.7, and polyethylene glycol tert-octylphenyl ether (Triton^TM^ X-100, laboratory-grade) were purchased from Sigma-Aldrich. The chemicals were purchased from the following suppliers: Sigma-Aldrich (St. Louis, MO, USA), Merck (Darmstadt, Germany), and EQUILAB (Santiago, Chile).

### 2.2. Synthesis of Ternary Bioglass (BG) Nanoparticles

The NPs were synthesized by the sol–gel method as described [26], resulting in a ternary molar composition of 58SiO_2_:40CaO:5P_2_O_5_. In brief, three solutions were prepared. The first solution was synthesized by dissolving 7.7 g of Ca(NO_3_)_2_∙3H_2_O in 117 mL of distilled water (0.3 M). Subsequently, a second solution was prepared by diluting 9.7 mL of TEOS in 63.5 mL of ethanol (0.7 M) and gradually adding it dropwise to the first solution while adjusting the pH to 1–2 using citric acid. The resulting mixture was then gradually introduced into a third solution containing 1.2 g of NH_4_H_2_PO_4_ dissolved in 1500 mL of distilled water (0.007 M), under vigorous stirring, adjusting the pH to 10 using 28% *w*/*v* NH_4_OH. The resulting combination was stirred for 48 h and aged for more than 48 h at room temperature. The precipitate was centrifuged (Biofuge 13, Heraeus Instruments, Hanau, Germany) and washed three times with distilled water. Then, the acquired solid was dispersed and stirred in 200 mL of a 2% *w*/*v* aqueous solution of PPG (Mw 2000 g/mol) for 24 h. Later, the suspension was freeze-dried at −80 °C for 2 h and then lyophilized (Christ Alpha 1–2 Freeze Dryer, Osterode am Harz, Germany) for 48 h. Finally, the obtained solid was calcinated (Oven Lindberg Blue M, ThermoFisher, Asheville, NC, USA) at 350 °C for 3 h.

### 2.3. Synthesis of ZnO Nanoparticles

The ZnO NPs were obtained using the sol–gel method reported by Castro et al. [27]. Two solutions were prepared; for the first solution, 5.5 g of ZnCl_2_ was dissolved in 200 mL (0.2 M) of water at 90 °C in a silicon bath with magnetic stirring at 750 rpm. The second consisted of an aqueous solution of 5.0 M of NaOH, of which 16 mL was added dropwise to the ZnCl_2_ solution with gentle stirring for 10 min at 90 °C in a silicon bath. The resulting mixture was allowed to cool at room temperature and left to separate by sedimentation until the next day. Later, the remaining suspension was washed and filtered (Whatman qualitative filter paper grade 1, Sigma Aldrich) thirteen times with distilled water, discarding the supernatant solution each time to lower the NaCl concentration below 10^−6^ M. The purified particles were peptized with isopropyl alcohol in an ultrasonic bath for 10 min at room temperature. The particles were collected by centrifugation (Biofuge 13, Heraeus Instruments, Hanau, Germany) at 5000 rpm for 15 min and then washed three times with isopropyl alcohol in a filter with a vacuum Kitasato flask and left to dry overnight in an oven (UF110 Plus, Memmert, Schwabach, Germany) at 60 °C. Finally, the particles were calcined in an oven (Lindberg Blue M, ThermoFisher, Asheville, NC, USA) at 250 °C for 5 h.

### 2.4. Preparation of PVA/SA with ZnO and BG Nanoparticles Blend Solutions

**Figure 1:**
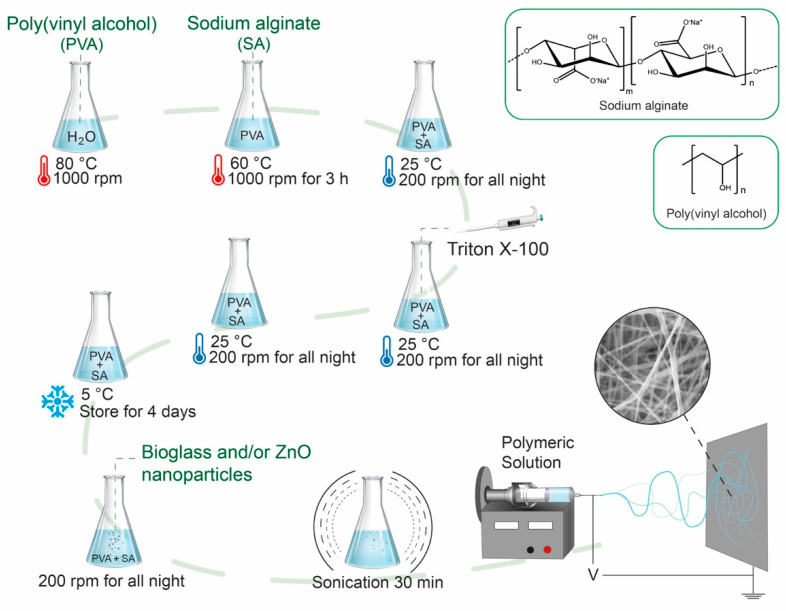
Schematic representation of electrospinning procedure of PVA/SA nanofibers containing ZnO and BG nanoparticles.

Based on preliminary tests, the polymer solution was optimized to 6.0 wt.% PVA and 0.8 wt.% sodium alginate, ensuring stable fiber formation during electrospinning. First, 600 mg of PVA was dissolved in 10 mL of water (6 wt.%), with magnetic stirring at 1000 rpm at 80 °C until a homogeneous mixture. The solution was left to cool until 60 °C, and then 80 mg of SA (0.8 wt.%) was added, with magnetic stirring at 1000 rpm at 80 °C for 3 h. Once the mixture was homogeneous, it was left to cool to ambient temperature and vigorously stirred all night. Later, 100 μL of Triton X-100 was added to the solution and stirred at 200 rpm all night. The resultant solution was incubated for four days at 5 °C. Subsequently, ZnO and/or BG NPs were added and stirred at 200 rpm all night. Table 1 summarizes the quantity of NPs used for the prepared solutions. Finally, the mixture was sonicated for 30 min in an ultrasonic bath (Elmasonic S60H, Elma Schmidbauer GmbH, Hohentwiel, Germany) for electrospinning. The electrospinning procedure is summarized in Figure 1.

### 2.5. Electrospinning of PVA/SA/ZnO/BG Blend Nanofibers

The previous solutions described in Section 2.4 were electrospun using electrospinning equipment (TL-01, Tong Li Tech, Shenzhen, China). Electrospinning was performed using a syringe (No. 2.3) connected to the needle via an alligator clip. A voltage of 21 kV was applied, and the solution was dispensed through a blunt needle tip using a syringe pump at a flow rate of 0.5 mL/h. Fibers were collected on electrically grounded aluminum foil with dimensions of 15 × 15 cm and placed at a 19 cm vertical distance from the needle tip.

### 2.6. Characterization

#### 2.6.1. ZnO and BG Nanoparticles

The synthesized NPs were studied by transmission electron microscopy (TEM) (Hitachi model HT7700, Tokyo, Japan). Size measurements on TEM images were carried out using the ImageJ 1.53c software. 100 NPs were randomly measured in the captured image, and the average NPs size for both ZnO and BG was calculated. In addition, dynamic light scattering (DLS) and ζ potential measurements were performed further to characterize the NPs’ hydrodynamic size and surface charge using a Zetasizer Advance (Malvern Panalytical, UK). For this, NPs samples were dissolved in ethanol and sonicated in a water bath for 45 min. NPs were also characterized through X-ray diffraction (XRD) (Bruker model D8 ADVANCE, Aubrey, TX, USA) using a Cu lamp (λ = 1.5418 Å) to evaluate the crystalline structure. The XRD patterns were obtained at room temperature at 10° < 2θ < 60°.

#### 2.6.2. PVA/SA/ZnO/BG/ Nanofibers

The morphology of the electrospun blends was conducted with a scanning electron microscope (SEM) equipped with energy-dispersive X-ray spectroscopy (SEM-EDS) (Zeiss model EVO MA10, Jena, Germany) after a gold coating in a Cressington 108 auto Sputter Coater (Cressington Scientific Instruments Inc, Cranberry Twp, PA, USA). Fiber and pore sizes were calculated and reported in this study. The mean and standard deviation were determined from SEM micrographs using Image J 1.53c software. The size distribution of fiber diameters and the average pore area was determined by measuring 100 randomly selected points in the SEM images obtained at a magnification of 10.00×.

Functional groups and crystallinity of nanofibers were characterized by Attenuated Total Reflectance Fourier-transform infrared spectroscopy (ATR-FTIR, Perkin Elmer model Spectrum Two, Waltham, MA, USA), and XRD (Bruker model D8 ADVANCE, Aubrey, TX, USA), respectively. Thermal behavior, such as melting temperature and enthalpy of fusion of the nanofibers, was measured by differential scanning calorimetry (DSC) (PerkinElmer model 4000, Columbus, OH, USA). The samples were heated from 0 °C to 350 °C at 10 °C/min. Approximately 5 mg of each sample was placed in 40 μL aluminum pans, with an empty pan serving as a control. All experiments were conducted in an inert nitrogen atmosphere with a constant flow rate of 2 mL/min. The melting temperature (T_m_) and melting enthalpy (ΔH_f_) were determined from the second heating. Percent crystallinity (X_c_) was determined using Equation (1).

(1) $$X_{C}=\frac{\Delta H_{m}\cdot 100}{\Delta H_{m}^{0}\cdot \left(1-\Phi \right)}$$

where ΔH_m_ is the melting enthalpy (J/g) of the scaffold, ΔH^0^_m_ is the value of the enthalpy corresponding to the melting of a 100% crystalline PVA (138.6 J/g), and Φ is the weight fraction of the NPs in the sample [28].

Thermal stability was characterized using thermogravimetric analysis (TGA) (PerkinElmer model TGA 4P0P, New Castle, DE, USA) under a nitrogen atmosphere with a 20 mL/min flux. Approximately 5 mg of each sample was weighed in a pan of 50 μL. Samples of nanofibers were heated from 25 °C to 800 °C at a heating rate of 10 °C/min.

#### 2.6.3. Mechanical Properties

The mechanical properties of nanofibers were studied using an Instron EMIC 23-5D(Norwood, MA, USA)at room temperature. Five membranes of each sample were cut into a 15 mm thick rectangular shape (10 mm × 50 mm) and dried in an oven at 35 °C for 24 h. The distance between the two chucks was 10 mm, and the stress–strain experiments were performed at a 1.0 mm/min speed. The mechanical properties of the developed nanofibers were determined in terms of Young’s modulus, tensile strength, and elongation at break [29].

### 2.7. In Vitro Antibacterial Evaluation

The antibacterial properties of the membranes were evaluated using the disc diffusion assay. The antimicrobial activity of the membranes and controls was evaluated against the strains *S. aureus* ATCC 25923, *E. coli* ATCC 25922, and *P. aeruginosa* ATCC 27853. A McFarland 0.5 suspension was prepared for each bacterial strain and subsequently diluted 1/10 and inoculated in Muller Hinton agar plates. As a screening, the initial tests employed 10 × 10 mm^2^ squared membrane cuts, which were sterilized by UV radiation for 30 min. Each membrane and control was incorporated into the Muller Hinton plates previously inoculated with the ATCC strains. Additionally, the antimicrobial activity of the membranes’ protective film was evaluated for any remaining residual activity. The plates were incubated for 18 h at 37 °C in a normal atmosphere. Subsequently, inhibition activity was observed. The next stage was conducted using only the strains of *Staphylococcus* spp. because it displayed an inhibition halo during the screening phase. Antibacterial activity was evaluated against *S. aureus* ATCC 25923, *S. epidermidis* ATCC 12228, and a clinical strain of methicillin-resistant *S. aureus* (MRSA) isolated from human wound secretion (HWS). In this phase, circle membranes were used, with a diameter of 18 mm. Membrane sterilization, inoculum preparation, bacterial inoculation, and incubation were conducted under the same conditions as in the initial test. After 18 h of incubation, the microbial inhibition zone was measured. Considering that the membranes decrease in size upon contact with the incubation medium, the diameter of the membranes post-incubation and the inhibition halo diameter were considered. The results were obtained by establishing a ratio between the inhibition halo and the post-incubation membrane size and then normalizing the results with respect to the control. The experiments were performed in quadruplicate.

### 2.8. In Vitro Cytotoxicity Evaluation

HaCaT cells, an immortalized human keratinocyte line, were cultured in 10 cm^2^ plates using Dulbecco’s Modified Eagle Medium (DMEM) containing 1.8 mM Ca^2+^ (Gibco, Invitrogen, ThermoFisher, Waltham, MA, USA). The medium was supplemented with 10% fetal bovine serum (FBS; Gibco), 2 mM L-glutamine (Gibco), 1% penicillin G-streptomycin (Gibco), and 1% Fungizone^®^ (Gibco). Cultures were maintained at 37 °C in a humidified incubator with 5% CO_2_. Once the cells reached 70–80% confluence, they were detached using 0.25% trypsin and 1 mM EDTA, then resuspended in fresh supplemented medium. A total of 6 × 10^6^ cells were seeded into a 12-well plate, followed by the addition of culture medium. When cell adhesion reached 70–80% confluence, a defined mass of scaffold material was introduced. Cell morphology and viability were evaluated over a 72-h period using bright-field microscopy. Upon completion of the incubation, cells were rinsed with 1X PBS, detached with 0.25% trypsin, and subsequently washed twice more with 1X PBS. For subsequent flow cytometric analysis, a total of 1 × 10^6^ cells were transferred into Eppendorf tubes, centrifuged for 5 min, and resuspended in 200 µL of flow cytometry staining buffer (eBioscence, San Diego, CA, USA). Propidium iodide (eBioscence, San Diego, CA, USA) was then added at a final concentration of 10 µg/µL, and the samples were incubated for 5 s. A control sample containing only staining buffer and PI was included for comparison. Samples were analyzed using a CytoFLEX Flow Cytometer (Beckman Coulter, Orange, CA, USA), with software settings configured to distinguish viable cells (dot scatterplot) from PI-stained ones. FlowJo V.10 software was used for data analysis. To prevent bias, all sample measurements were conducted in a blinded manner [30].

### 2.9. Statistical Analysis

A 2^3^ factorial design was performed with two qualitative levels (type of NPs, ZnO, and BG) and three quantitative levels (NPs concentration, 5 and 10 wt.%, and ZnO/BG combination). An analysis of variance (two-way ANOVA) was conducted to determine statistically significant differences between groups, followed by Bonferroni’s test. The values of * = *p* > 0.05, ** = *p* > 0.01, and *** = *p* > 0.001 were considered significant.

## 3. Results and Discussion

### 3.1. ZnO and Bioglass Nanoparticles Characterization

**Figure 2:**
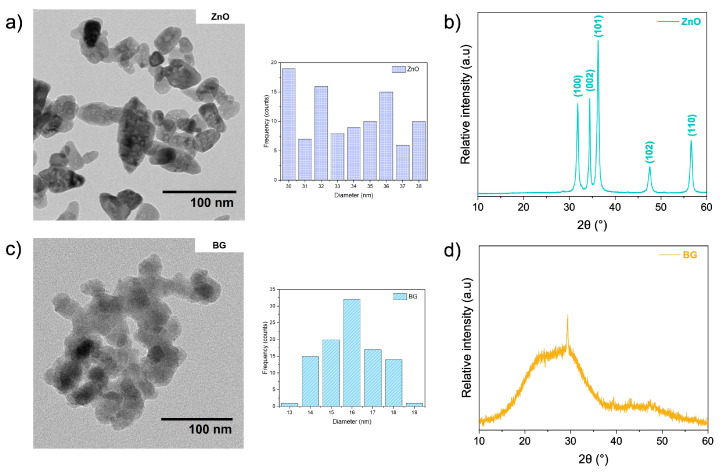
TEM images and histograms of (**a**) ZnO and (**c**) BG NPs obtained via the sol–gel method. XRD pattern of (**b**) ZnO and (**d**) BG NPs.

The TEM image of ZnO NPs (Figure 2a) shows an oval-shaped morphology with an average diameter of 33 ± 3 nm. Furthermore, the Bragg reflections (Figure 2b) were in a 10–60° range with diffraction peaks (2θ) at 31.8°, 34.4°, 36.3°, 47.5°, and 56.6° corresponding to planes (100), (002), (101), (102) and (110), respectively, which is characteristic of hexagonal wurtzite phase of ZnO (JCPDS, card number 36-1451) [31,32]. Figure 2c displays the TEM image of bioglass (BG) NPs, which exhibit a spherical-like morphology with about 15 ± 1 nm diameters. Figure 2d shows the characteristic broad and low-intensity diffraction peak of the BG amorphous structure between 15° and 35° due to the amorphous SiO_2_ glass network [33]. The experimental elemental composition obtained by SEM-EDS analysis was 54SiO_2_:40CaO:6P_2_O_5_, as previously reported by our research group [26]. On the other hand, the hydrodynamic sizes obtained for ZnO and BG were 471.7 ± 15.0 nm and 118.9 ± 10.5 nm, respectively. These average values, which are higher than those found through individual counting in TEM, can be attributed to the formation of aggregates that could not be disaggregated during the sonication time, as also observed in the TEM images (Figure 2). The measured ζ potential values, 7.083 ± 0.448 mV (ZnO) and −4.769 ± 0.604 mV (BG), support the same conclusion, as their relatively low values indicate low-to-poor colloidal stability in aqueous solution. Notably, similar size distributions and surface charge characteristics have been reported in previous studies for both types of NPs, suggesting that these behaviors are inherent to their physicochemical nature [34,35].

### 3.2. Preparation and Morphology of PVA/SA Nanofibers with ZnO and BG Nanoparticles

**Figure 3:**
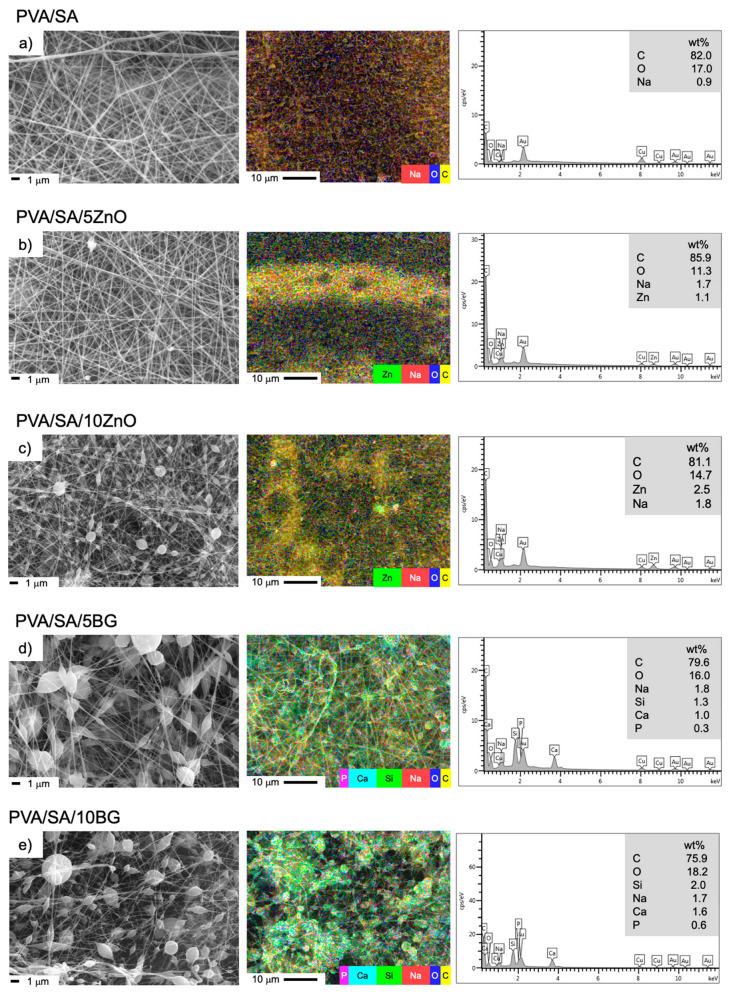
SEM images, elemental mapping distribution, and EDS spectra of PVA/SA membranes containing 5–10 wt.% of ZnO or BG (54SiO_2_:40CaO:6P_2_O_5_) NPs, (**a**) PVA/SA, (**b**) PVA/SA/5ZnO, (**c**) PVA/SA/10ZnO, (**d**) PVA/SA/5BG, (**e**) PVA/SA/10BG.

**Figure 4:**
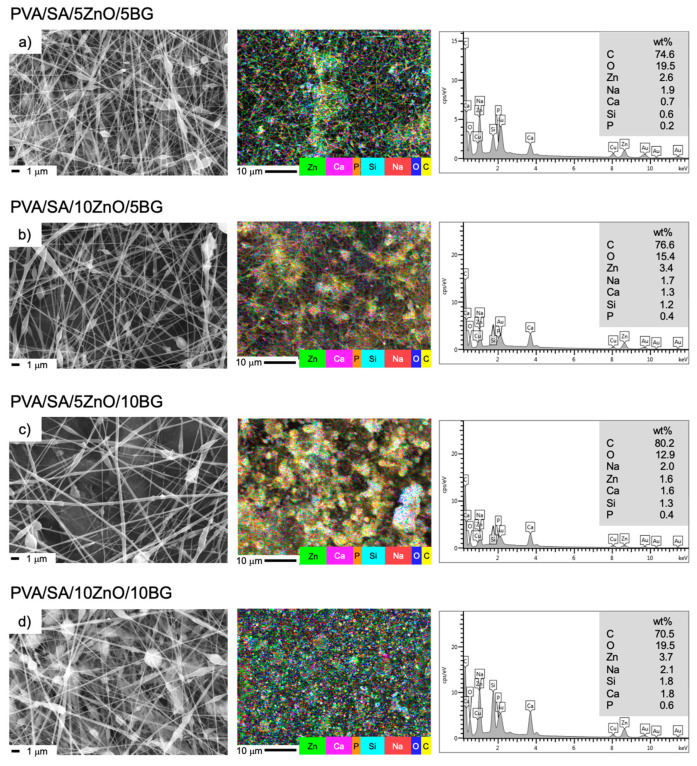
SEM images, elemental mapping distribution, and EDS spectra of PVA/SA membranes containing a combination of different weight ratios of ZnO and BG (54SiO_2_:40CaO:6P_2_O_5_) NPs, (**a**) PVA/SA/5ZnO/5BG, (**b**) PVA/SA/10ZnO/5BG, (**c**) PVA/SA/5ZnO/10BG, (**d**) PVA/SA/10ZnO/10BG.

The electrospinning of a SA solution is complex due to its chain conformation and high repulsive force. By introducing PVA as a synthetic neutral macromolecule, this repulsion is reduced, improving the solution’s spinnability [36]. Figure 3 and Figure 4 show the SEM images, elemental mapping distribution, and EDS spectra of the PVA/SA composites and PVA/SA/ZnO/BG membranes, respectively. In general, it was found that the electrospinning technique produces an interconnected fibrous network with a well-defined morphology, randomly distributed with spaced and homogeneous fibers based on the PVA/SA matrix. Several reports indicate that this type of topography is of great interest because this range of nanofiber diameter, interconnectivity, and high surface area mimics the extracellular matrix architecture and is conducive to structural support for cell growth, skin cell viability, adhesion, and proliferation [37,38]. In addition, the porosity of the membranes supports the transport of nutrients and oxygen, facilitates the removal of cellular waste, enables cell migration, and promotes the repair of damaged skin tissue.

**Figure 5:**
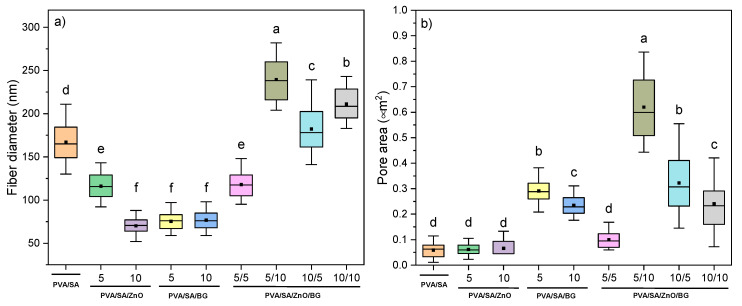
(**a**) Average diameter and (**b**) pore area of PVA/SA nanofibers containing 5–10 wt.% of ZnO and BG NPs. Means that do not share a letter differ significantly (n = 100, *p* ≤ 0.0001).

The average diameter of fibers and pore area from PVA/SA-based nanofibers are shown in Figure 5, Figure S1, and Table S1. The fiber diameters of all formulations are in the range of ca. 80–240 nm, and the pore area is between ca. 0.06 and 0.60 μm^2^. PVA/SA/ZnO membranes have a significantly lower fiber diameter than PVA/SA (*p* ≤ 0.0001), which is ca. 42% lower in PVA/SA10ZnO membranes compared to PVA/SA, as previously reported for PVA [39], PVA/SA/graphene oxide [40], and PVA/polyvinylpyrrolidone [41] nanofibers. This may be attributed to the fact that the ZnO NPs added in high concentrations increased the viscosity and electrical conductivity of the solution compared to the neat PVA/SA solution, causing the shrinkage of the fibers [39,42]. Regarding pore size, this did not experience significant changes in the PVA/SA/ZnO membranes. Besides fiber diameter, the morphology changes noticeably upon the addition of ZnO NPs, and beads are observed as part of the fibers of variable size and distribution. These spheres increase in size and frequency with increasing ZnO NPs concentration due to NPs agglomeration, which could be attributed to the weak interaction between the PVA/SA matrix and the ZnO NPs and the low ζ potential of ZnO NPs.

With the incorporation of 5 and 10% BG NPs in the PVA/SA matrix, the same effect as the incorporation of ZnO NPs in the PVA/SA matrix is observed; fiber diameter decreases significantly by ca. 55%, and the BG NPs tend to agglomerate as elongated spheres at both concentrations. However, the increase in BG NPs concentration does not lead to a statistically significant effect on the fiber diameter, although the pore size of PVA/SA/BG fibers significantly increased about five times concerning PVA/SA. The same trend and similar morphology were previously reported in the elaboration of PVA nanofibers filled with 5–40 wt.% of BG NPs (based on Si, Ca, and P) [43]. On the contrary, the addition of 45S5 bioglass (45% SiO_2_, 24.5% CaO, 24.5% Na_2_O, and 6.0% P_2_O_5_), between 1 and 3 wt.% in PVA/gelatin membranes, was found to be free of beads and defects [12].

The elemental composition of PVA/SA, PVA/SA/ZnO, and PVA/SA/BG membranes was analyzed using SEM-EDS (Figure 3). The EDS spectra indicate that all membranes contain C and Na, as they are composed of PVA and sodium alginate (SA) polymers. Additionally, the PVA/SA/ZnO membranes show a proportional increase in Zn content with the increasing ZnO NPs load. A similar trend is noticed in PVA/SA/BG membranes, where an increase in Si, Ca, and P, attributed to the chemical composition of BG NPs (54SiO_2_:40CaO:6P_2_O_5_), is observed. Moreover, elemental mapping demonstrates a generally uniform distribution, confirming the constituent elements of the loaded NPs within the electrospun matrices.

The ternary PVA/SA/ZnO/BG membranes (Figure 4 and Figure 5) generally exhibited a significant increase in fiber diameter, reaching up to 45% compared to PVA/SA. It is suggested that the increasing concentration of ZnO and BG NPs leads to a proportional rise in solution viscosity. According to Koricka et al. [44], the dynamic viscosity of the feed solution has the most significant impact on the diameter of beaded fibers and beads. While the combination of both NPs did not show a clear trend in pore size, it had a notable effect on the pore structure of PVA/SA. The morphology of the PVA/SA/ZnO/BG nanofibers closely resembles that of the PVA/SA/BG membranes (Figure 3), likely due to the dominant influence of BG NPs. This effect is especially noticeable in forming larger beads, indicating that BG NPs are crucial in shaping fiber morphology and bead size. In addition, the number of elongated spheres is appreciably lower, suggesting a good integration between metal oxide NPs and the PVA/SA matrix, which agrees with the increase in fiber size. This topography has also been reported recently for poly(L-lactic acid) scaffolds containing ternary ZnO and BG NPs, both at 10 wt.% [24], while the increase in fiber size, with an absence of aggregations, was obtained in poly(caprolactone)/ZnO/45S5 BG membranes at a weight ratio 8:1:1 [25].

Regarding the elemental composition of PVA/SA/ZnO/BG membranes (Figure 4), a uniform distribution of all elements constituting the PVA and SA polymers and the ZnO and BG NPs is observed. Additionally, a clear correlation between the NP content and the elemental composition is observed, confirming the successful incorporation of these NPs.

### 3.3. Characterization of PVA/SA Nanofibers with ZnO and BG Nanoparticles

#### 3.3.1. ATR-FTIR Spectroscopic and XRD Characterization

**Figure 6:**
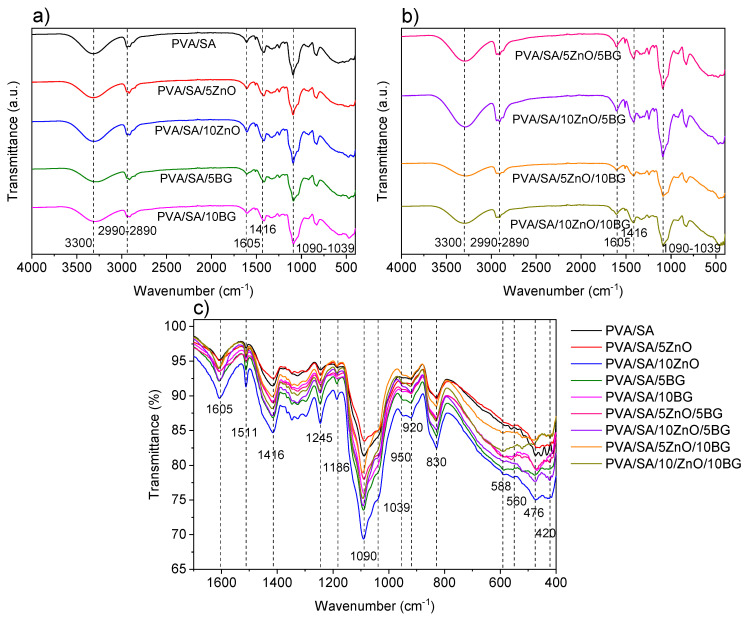
ATR-FTIR spectra in the region 4000–400 cm^−1^ of PVA/SA membranes containing (**a**) 5–10 wt.% of ZnO or BG NPs, and (**b**) combination of different weight ratios of ZnO and BG NPs. (**c**) Superposed ATR-FTIR spectra in the 1700–400 cm^−1^ region.

Figure 6 shows the qualitative analysis by ATR-FTIR spectroscopy of PVA/SA membranes with ZnO and BG NPs. A broad absorption band centered at ca. 3230 cm^−1^ is observed in the spectra of all nanofibers, which is representative of the ν O-H of the hydroxyl groups of both PVA and SA, and silanol groups (Si–OH). In the 2990–2890 cm^−1^ region, the signals are attributed to the νs/νas vibrational modes of both polymer chains, corresponding to the C-H bond. The medium intensity signals at 1605 and 1416 cm^−1^ are attributed to the ν_as_ and ν_s_ of the COO- groups of SA, respectively [45]. The signal at 1416 cm^−1^ is also assigned to the contribution of the δ C-H of the PVA backbone. The low-intensity signals at 1511 and 1245 cm^−1^ are due to the δ C-O-H and δ C-H vibrational modes of the SA, while a set of low-intensity signals between 1367 and 1294 cm^−1^ corresponds to the δ O-H, rocking, and C-H wagging, and δ C-O-H of both polymers. The spectra exhibit a low-intensity signal at 1186 cm^−1^ attributed to ν C-O of PVA and SA, with the influence of δ C-O-H; in addition, two prominent and robust bands at 1090-1039 cm^−1^ are assigned to ν_as_ C-O-C of glycosidic linkage vibration with contributions of ν C-O (pyranosyl ring) and δ C-C-C-O vibrations of SA [46,47]. The 950–700 cm^−1^ region corresponds to the carbohydrate fingerprint or anomeric region of SA. The signal at 950 cm^−1^ is attributed to the C-O stretching vibration of uronic acid residues of SA, and the band at 830 cm^−1^ is due to the anomeric ν C-H of polymannuronic acid (MM blocks) with the contribution of the ν C-C of PVA [45]. The low-intensity signal at 920 cm^−1^ is assigned to the δ O-H and δ C-C-C vibrational modes.

In turn, distinctive signals stand out at lower wavenumbers in the NPs-loaded membranes. In the ATR-FTIR spectra of the membranes containing ZnO NPs, new signals concerning PVA/SA at 588, 476, and 420 cm^−1^ are assigned to the stretching and deformation vibration modes of the Zn-O bond [32,39]. Regarding membranes with BG NPs, a new signal at 560 cm^−1^ is attributed to the stretching of the P-O bond [48]. Other characteristic BG signals at 1240 and 800 cm^−1^ corresponding to the ν_as_ and ν_s_ of the Si-O-Si groups, respectively, and 580 cm^−1^ Si-O bending, are overlapped by the constituent functional groups of the PVA/SA matrix [26,49].

**Figure 7:**
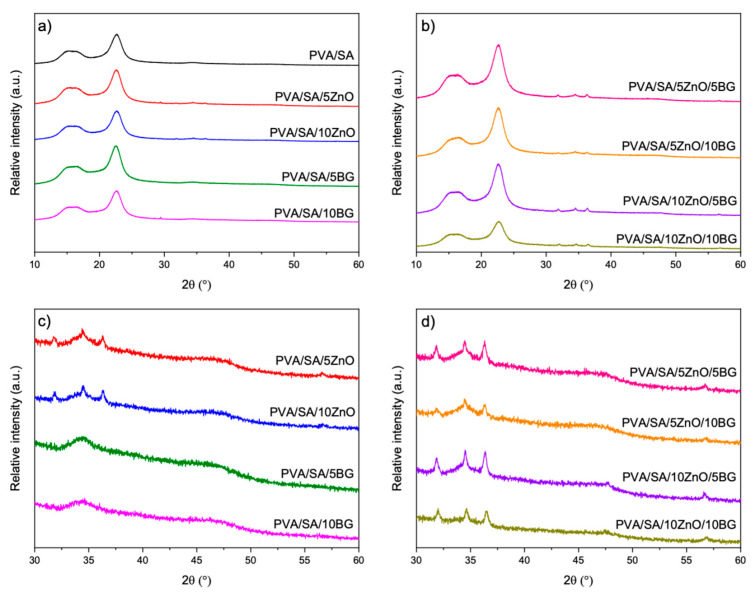
XRD of electrospun fibers of PVA/SA and PVA/SA membranes containing (**a**) 5–10 wt.% of ZnO or BG NPs, and (**b**) a combination of different weight ratios of ZnO and BG NPs. (**c**,**d**) Amplified XRD patterns in the range of 2Θ 30–60°.

The crystalline structure of PVA/SA membranes and their binary and ternary composites was analyzed by XRD, as shown in Figure 7. The diffraction patterns of all membranes show two peaks at 15.9° and 22.5°, related to the (110) plane of the polygalacturonic acid (GG blocks) of SA [50] and semicrystalline PVA [51], respectively. It can also be noticed that the presence of ZnO and BG NPs did not alter the observed pattern of the PVA/SA matrix. In the XRD diffractogram of the membranes containing ZnO NPs, low-intensity diffraction peaks at 31.8°, 34.4°, 36.3°, 56,6° are observed corresponding to the planes (100), (002), (101), (102), and (110), respectively, of the hexagonal wurtzite phase of ZnO NPs. In the diffractograms of the membranes containing BG NPs, no new peaks are distinguished from the matrix without NPs because the BG NPs are constituted of an amorphous SiO_2_ glass network (Figure 2). Therefore, these results verify the successful PVA/SA membranes formulation filled with ZnO and BG NPs.

#### 3.3.2. Thermal Properties

The thermal stability of electrospun PVA/SA and PVA/SA membranes with NPs was studied by TGA (Figures S2 and S3). The TGA analysis of membranes indicates a mass loss of ca. 5% at ca. 90 °C due to loss of absorbed water. Subsequently, two thermal decomposition zones of the fibers are exhibited, the first between 200 and 275 °C and the second between 290 and 470 °C. It corresponds to the partial depolymerization temperature and the final degradation temperature of PVA/SA, respectively [52]. The temperature at when the membranes lost 10% of their weight (T_10_) and the maximum degradation temperature (T_max_) were analyzed (Table 2). The incorporation of ZnO NPs did not change the T_max_ of PVA/SA membranes, as reported by Shalumon [53], while BG NPs increased the T_max_ to about 6 %. The same trend was obtained for PVA/SA/ZnO/BG membranes, which is attributed to the higher thermal stability of BG NPs [49]. 

The thermal behavior of the fibers was further evaluated using differential scanning calorimetry (DSC) (Figures S4 and S5). The glass transition temperature (T_g_), cold crystallization temperature (T_cc_), melting temperature (T_m_), and crystallinity percentage (X_c_) are shown in Table 2. The results show that the T_g_ did not considerably change with the type and concentration of NPs incorporation. In the DSC cooling curves (Figure S5), no exothermic peaks associated with the T_cc_ of PVA/SA and PVA/SA/ZnO fibers were detected. However, BG NPs are observed to reduce the T_cc_ of the neat PVA membrane, which is approximately 178 °C (see Figure S5). This reduction is suggested to be due to their ability to distribute heat more evenly and induce supercooling, which accelerates heterogeneous nucleation. Additionally, BG NPs serve as nucleation sites for PVA crystallization; however, their strong interactions with PVA shorten the crystallization process. Adding BG NPs to electrospun fibers shifts T_cc_ to a higher temperature and decreases the crystallization enthalpy due to the reduction of conformational entropy from the preferential orientation of the polymer chains [54].

In addition, from the characterization of the fibers using DSC, it is observed that the T_m_ and X_c_ of composites show variations compared to PVA/SA. In the case of ZnO NPs, a proportional decrease in the crystallinity of the polymer matrix is observed as the filler content increases. This is associated with a disruption of the crystalline arrangement of PVA, and as a result, the crystalline domains are melted at lower temperatures. A recent study reported that in an electrospun matrix of PVA/sodium caseinate, adding 2–6 wt.% of ZnO NPs slightly decreased the T_m_ of the electrospun fibers [55].

A different trend is observed regarding the fibers containing BG NPs compared to those with ZnO NPs, as BG NPs increase the fibers’ crystallinity. This indicates that, unlike ZnO NPs, BG NPs promote the formation of more crystalline regions within the matrix, likely due to their more uniform dispersion [56]. These findings are consistent with the SEM analysis (Figure 3 and Figure 4) and the greater thermal stability of BG NPs compared to ZnO NPs, as observed through TGA. Therefore, melting the PVA/SA and BG NP-based fibers requires more energy than the pure matrix. Similar results to those obtained in the present study were reported in the production of electrospun fibers of PVA/BG 58S (58SiO_2_-33CaO-9P_2_O_5_) with NPs loads in the range of 5–30 wt.% [57].

#### 3.3.3. Mechanical Properties

**Figure 8:**
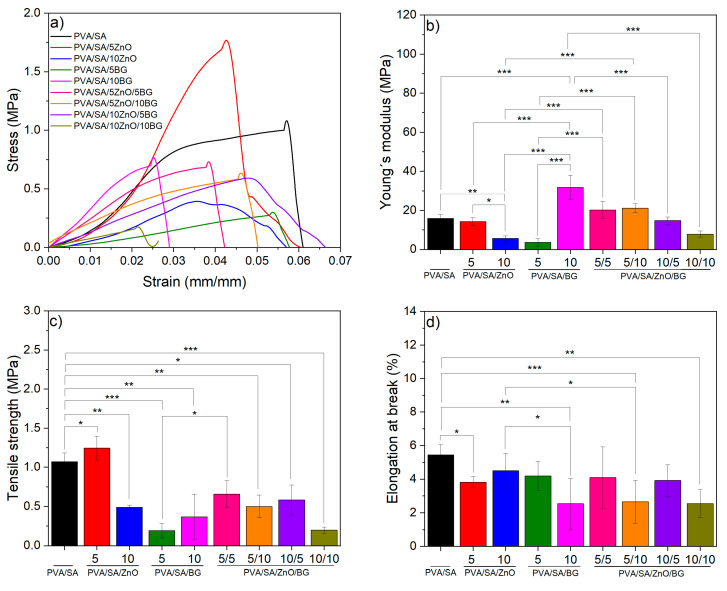
Mechanical properties of PVA/SA and PVA/SA nanofibers with ZnO and BG NPs. (**a**) Stress–strain curves, (**b**) Young’s modulus, (**c**) tensile strength (TS), and (**d**) elongation at break (n = 6, * = *p* < 0.05, ** *p* < 0.01, *** = *p* < 0.001).

The mechanical performance of the membranes and the influence of the NP loading were studied by evaluating the material’s stiffness, the maximum stress it could withstand during stretching, and its ability to deform before breaking. These were determined by measuring Young’s modulus, tensile strength (TS), and elongation at break, respectively (Figure 8 and Table S2). 

The results indicate that pure PVA/SA membranes exhibit a Young’s modulus of 15.9 ± 2.0 MPa. However, the incorporation of 10 wt.% ZnO nanoparticles resulted in a significant 65 wt.% reduction in Young’s modulus, with a tendency to further decrease as the ZnO nanoparticle content increases. In contrast, loading the membranes with 10 wt.% BG NPs resulted in a significant increase of 100% in Young’s modulus compared to that of the PVA/SA membrane. No significant variation in the modulus was observed in the membranes composed of both types of NP. Regarding tensile strength and elongation at break, the PVA/SA membranes exhibited values of 1.1 ± 0.1 MPa and 5.4 ± 0.6%, respectively, and the results show that incorporating NPs decreases these values compared to the pure matrix.

The dependence on mechanical properties can be correlated not only with the filler but also with the fibrillar morphology, its porosity, and the fiber diameter of the membranes. Studies have shown that nanofillers improve mechanical properties in homogeneous fibers with appropriate nanofiller integration or those free from defects or beads. Chen et al. studied the effect of BG NPs on the mechanical properties of chitosan/PVA/BG membranes, demonstrating that BG NPs at 5 and 10 wt.% increased yield strength and breaking strength but reduced elongation. However, a high nanofiller content at 20 and 40 wt.% significantly decreased both TS and elongation due to stress concentration points caused by the larger amount of BG NPs, which weakened mechanical properties [43]. 

A decreasing fiber diameter has also been demonstrated to improve molecular orientation, leading to enhanced mechanical properties [58]. However, our results generally show the opposite correlation when fiber diameter decreases, as higher nanofiller concentrations weaken mechanical properties. This is attributed to the bead-like fiber morphology and low uniformity, which reduces the bonding within the matrix. Consequently, there was a loss of friction during load transfer between the fibers, counteracting the reinforcement effect of the increased NPs content and compromising the mechanical properties [58].

Despite the above, the ternary PVA/SA/ZnO/BG membranes obtained in this study exhibit mechanical properties comparable to commercial wound dressings based on SA, such as Kaltostat™ (TS 0.9 MPa and elongation 10.8%) and Integra™ (TS ≥ 0.63 MPa, elongation 16%) [59], which consists of a bovine tendon collagen/glycosaminoglycan matrix. Therefore, the PVA/SA/ZnO/BG membranes demonstrate adequate mechanical strength for wound dressing applications.

### 3.4. In Vitro Antibacterial Evaluation

**Figure 9:**
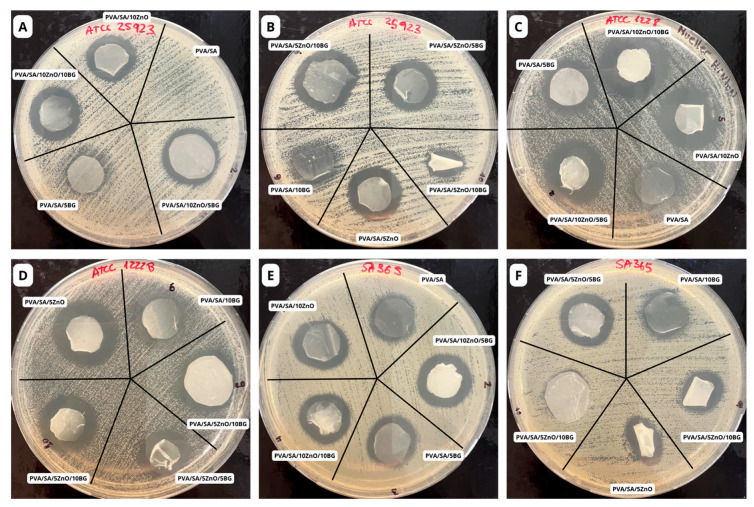
Representative antibacterial zone inhibition of PVA/SA membranes with ZnO and BG NPs against (**A**,**B**) *S. aureus* ATCC 25923, (**C**,**D**) *S. epidermidis* ATCC 12228, and (**E**,**F**) clinical methicillin-resistant *S. aureus* (MRSA) strains isolated from a human wound secretion (HWS).

**Figure 10:**
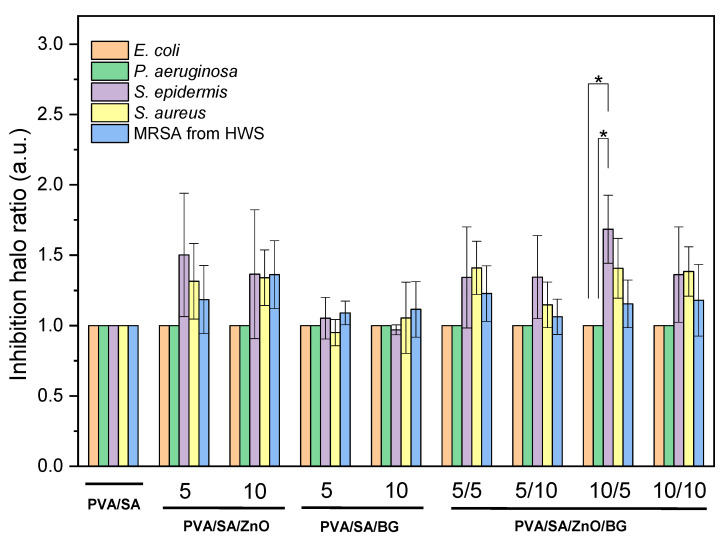
In vitro antibacterial activity of PVA/SA and PVA/SA nanofibers with ZnO and BG NPs against *E. coli*, *P. aeruginosa*, *S. epidermis*, *S. aureus*, and clinical methicillin-resistant *S. aureus* (MRSA) isolated from a human wound secretion (HWS) (n = 4, * = *p* < 0.05). The values are presented as the ratios for inhibition halo diameters normalized against the diameters of pure polymer membranes.

Figure 9 and Figure 10 show the in vitro antibacterial activity against Gram-negative and Gram-positive pathogenic bacteria measured through the inhibition halo ratio. None of the membranes exhibited the ability to reduce the growth of the Gram-negative bacteria *E. coli* and *P. aeruginosa*, which is attributed to the structural composition of these bacteria, including an additional outer membrane composed of lipopolysaccharides and porins that hinders the permeation of bactericidal compounds [60]. Although ZnO NPs are well-known antibacterial fillers against *E. coli*, their effectiveness depends on concentration. No antibacterial effect was observed in PCL fibers containing 4 wt.% ZnO NPs, whereas higher concentrations exhibited antibacterial activity against *E. coli* [61]. Moradi et al. also reported no antibacterial activity against *E. coli* in vitro with silk fibroin/SA/polyethylene oxide membranes containing less than 10 wt.% ZnO NPs. However, they observed a 2 mm inhibition zone when the ZnO NPs content was increased to 15 wt.% [21].

In turn, all composited membranes exhibited antibacterial activity against Gram-positive *S. epidermidis*, *S. aureus*, and clinical strain MRSA isolated from human wound secretion (HWS). In general, it is noticed that ZnO NPs lead to greater inhibition of Gram-positive bacteria in both binary and ternary composites than binary composite membranes composed solely of BG NPs. Antibacterial performance in increasing order was observed against MRSA, *S. aureus*, and *S. epidermidis* strains, with the latter achieving a ca. 68% greater inhibition than that of the PVA/SA membranes. These results show the potential of these composites, as *S. aureus* and MRSA from HWS strains are common bacteria in infected soft tissues and, despite being usually a harmless symbiotic bacterium, *S. epidermidis* can become pathogenic once it enters the human body [62]. Indeed, *S. aureus* is a versatile pathogen that can cause various diseases in humans, and MRSA is a significant threat to global public health due to high virulence and rapid spread [63,64].

It has been hypothesized that the antibacterial activity of BG results from an increase in local pH, caused by the exchange of sodium ions with protons in bodily fluids [65]. This shift to a more alkaline environment stresses bacteria, leading to changes in their morphology, ultrastructure, and the expression of various genes and proteins. Furthermore, the release of silica, calcium, and phosphate ions disrupts the membrane potential of bacteria and increases osmotic pressure, contributing to the antibacterial effect [65].

According to Mendes et al., the bactericidal mechanism of action of ZnO NPs is attributed to their ability to attach to the surfaces of Gram-positive and Gram-negative bacteria through various pathways [66]. The negatively charged bacterial cell surface, primarily due to teichoic acid in the peptidoglycan layer and lipoteichoic acid in the cell wall, electrostatically attracts the positively charged ZnO NPs. This interaction leads to surface damage. Furthermore, teichoic and lipoteichoic acids function as chelating agents for Zn^2+^ ions, facilitating their passive diffusion through membrane proteins. Bactericidal action can also occur through different mechanisms, such as adsorption on the bacterial surface and formation of various intermediates [66]. Therefore, these findings underscore the potential of PVA/SA/ZnO/BG membranes to prevent or treat infections in damaged skin, including abrasions, open wounds, and chronic wounds, as an alternative to conventional antibiotics. Recently, Duygulu et al. reported an inhibition halo of 8.1 mm against *S. aureus* with electrospun PVA membranes containing 3 wt.% ZnO NPs, smaller than the PVA control (13.5 mm) [67]. Canales et al. reported a similar trend to the present study in PLA/BG/ZnO cytocompatible membranes, with antibacterial effects primarily attributed to the ZnO NPs content [24]. Another study reported that chitosan/PVA/ZnO nanofibrous membranes improve wound healing capacity in diabetes-induced rabbits. Furthermore, the inhibition zones of chitosan/PVA/ZnO nanofibrous membranes against *E. coli*, *P. aeruginosa*, and *S. aureus* were 20.2 ± 1.0, 21.8 ± 1.5, and 21.5 ± 0.5 mm, respectively, compared to 14.1 ± 0.8, 15.8 ± 1.0, and 5.4 ± 0.5 mm for neat chitosan/PVA [68]. 

While previous studies have explored ZnO or BG NPs in electrospun systems—typically using PLA, PCL, or Gel matrices—few have combined both components as separate phases within a natural/synthetic scaffold. For example, Temel-Soylu et al. developed PVA/gelatin nanofibers incorporating BG but without ZnO or antibacterial evaluation [12]. Rajzer et al. used Zn-doped BG in PCL membranes for nasal regeneration, but did not assess antibacterial activity, and incorporated zinc as a dopant within the glass matrix rather than as a separate nanoparticle phase [69]. Moreover, Canales et al. reported PLA-based scaffolds with ZnO and BG NPs for bone repair, including antibacterial and cytocompatibility studies [24]. Our study advances these approaches by evaluating the combined effect of sol–gel–derived BG and ZnO NPs—each as distinct phases—incorporated into electrospun PVA/SA membranes. Notably, this study reports antibacterial activity not only against standard Gram-positive strains but also against methicillin-resistant *Staphylococcus aureus* (MRSA), a major clinical concern in chronic wound infections. This biofunctionality supports the potential of the developed system as a wound dressing for complex wound management.

### 3.5. In Vitro Cell Viability and Cytotoxicity Evaluation

**Figure 11:**
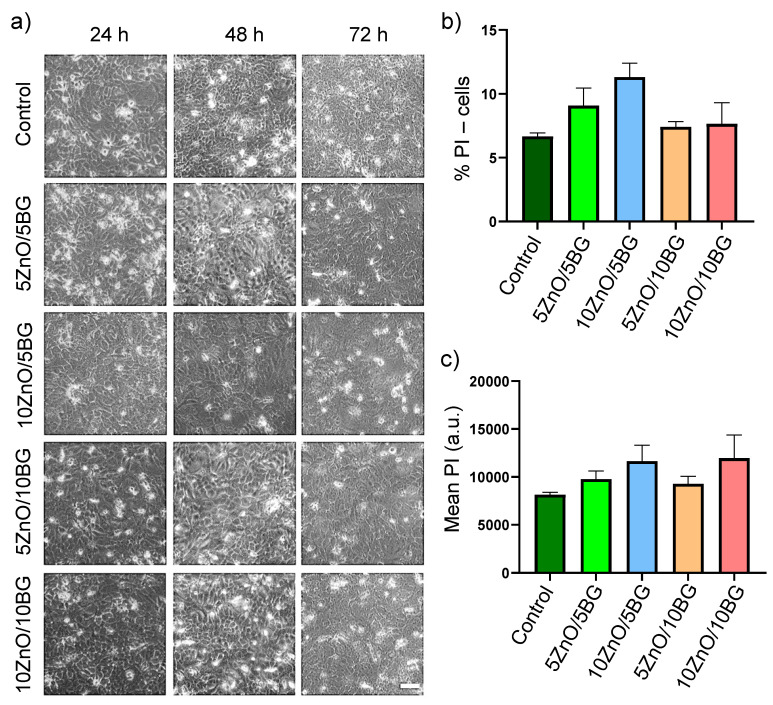
Cytotoxic evaluation of PVA/SA and PVA/SA/ZnO/BG membrane prototypes in HaCaT cells after 72 h. The cytotoxicity and cell viability of PVA/SA membranes, both unloaded and loaded with 5–10 wt.% ZnO and BG NPs were assessed in HaCaT cells at 24, 48, and 72 h of exposure. (**a**) Bright-field microscopy images (scale bar: 100 μm) showed no morphological changes in HaCaT cells following treatment. (**b**) Percentage of propidium iodide (PI)-positive (dead) cells after 72 h. (**c**) Mean fluorescence intensity of intracellular PI was measured after 72 h. No statistically significant differences were observed (n = 3).

Fibrillar membranes must not only have a high surface area, enable gas exchange, and absorb exudate through their porous matrix but also protect damaged skin from pathogenic bacteria during healing [70]. Additionally, they should be non-toxic and interact safely and beneficially with skin cells without triggering any harmful response. In vitro cytotoxicity was measured in an immortalized human keratinocyte cell line (HaCaT) to assess the potential of fibrillar membranes as wound dressing devices. Keratinocytes, which comprise 95% of the epidermis, play essential roles in maintaining skin structure barrier functions, the inflammatory response, and skin repair [71]. Therefore, keratinocytes were selected to assess the in vitro cellular response to the membranes. Figure 11 presents the viability of HaCaT cells after up to 72 h of incubation with the PVA/SA and PVA/SA/ZnO/BG membranes. The tertiary composites were studied for their potential angiogenic [11] and antibacterial properties [14], ensuring their cytocompatibility and safety in infected chronic wounds. No evident signs of toxicity were detected in HaCaT cells after 72 h of exposure to the membranes. Bright-field microscopy images (Figure 11a) reveal the preservation of cell population morphology across all PVA/SA/NPs formulations and periods. Propidium iodide (PI) was used to stain HaCaT cells at the end of the treatment period (72 h), with fluorescence intensity analyzed via flow cytometry. PI is widely employed to evaluate cell viability due to its ability to penetrate compromised membranes and intercalate with double-stranded DNA. The PI-positive cell population, indicative of cell death (Figure 11b), along with the mean PI incorporation in HaCaT cells (Figure 11c), showed no significant differences in the presence of PVA/SA/NPs membranes. These findings suggest minimal to no cytotoxicity.

The literature reports that controlled concentrations of ZnO NPs can promote cell growth, proliferation, and differentiation, along with supporting tissue regeneration by enhancing angiogenesis and osteointegration. ZnO’s antibacterial and antifungal properties further reinforce these benefits. However, ZnO NPs can also induce toxic effects in cellular environments, resulting from cell membrane damage, Zn^2+^ ion release, and the generation of reactive oxygen species (ROS) [72]. In this regard, Canales et al. designed PLA/ZnO/BG membranes with 20 wt.% NP filler using the electrospinning technique and evaluated cell viability with ST-2 bone marrow cells after 1 and 7 days. They reported a decrease in cell population at high concentrations of ZnO NPs in PLA/ZnO membranes. However, this cell death was partially counteracted by adding BG NPs, concluding that cytotoxicity can be modulated by incorporating bioceramic NPs [24]. In another study, an improvement in cell viability was observed after 48 h with electrospun PCL/ZnO/BG membranes (8:1:1 wt ratio) compared to PCL/BG when tested against MG-63 cells (human osteoblast-like cells). The authors attributed this improvement to the fact that Zn ions are essential dietary supplements that act as cofactors for numerous enzymes and assist in cell signaling pathways, which may have promoted cell growth and enhanced the cell adhesion properties of the PCL/BG membranes. Additionally, the authors reported that adding Ag NPs to the PCL/BG membranes reduced cell viability by 52% [25].

Further studies are needed to fully validate the biocompatibility of PVA/SA/ZnO/BG membranes. However, the biomimetic prototypes developed in this study exhibit no signs of cytotoxicity, making them promising cytocompatible candidates for wound dressings that support healing and provide protection against bacterial infections.

## 4. Conclusions

Nanofibrous membranes composed of PVA, sodium alginate, and therapeutic NPs were successfully developed, exhibiting cytocompatibility and attractive antibacterial properties for wound dressing applications. The electrospinning technique enabled the formation of an interconnected nanofibrous network with uniformly distributed fibers (diameters between 80 and 240 nm) and homogeneous NPs dispersion within the PVA/SA matrix. This architecture closely mimics the natural extracellular matrix, supporting cellular functions critical for skin repair and regeneration.

The prototyped membranes demonstrated mechanical properties comparable to commercial wound dressings. The incorporation of ZnO and BG NPs into PVA/SA membranes significantly influenced their mechanical properties. Pure PVA/SA membranes showed a Young’s modulus of 15.9 ± 2.0 MPa, tensile strength of 1.1 ± 0.1 MPa, and elongation at break of 5.4 ± 0.6%. Adding 10 wt.% ZnO reduced stiffness by 65%, while 10 wt.% BG increased it by 100%. Combined ZnO/BG formulations maintained intermediate stiffness. Despite a decrease in tensile strength and flexibility across all NP-loaded membranes, the tunable mechanical performance supports their suitability for tailored wound dressing applications.

Notably, the membranes loaded with ZnO and BG NPs exhibited antibacterial activity against the ATCC strains *Staphylococcus epidermidis*, *Staphylococcus aureus*, and clinical methicillin-resistant *Staphylococcus aureus* (MRSA) strains isolated from human wound secretions. This antimicrobial effect was mainly attributed to the presence of ZnO NPs. The ability to inhibit MRSA is particularly relevant, as this pathogen is a leading cause of chronic and hospital-acquired wound infections worldwide, with increasing prevalence due to antibiotic resistance.

Furthermore, the membranes preserved human keratinocyte viability after 72 h, as confirmed by both fluorescence and flow cytometry, indicating a high cytocompatibility and absence of toxicity. These findings support the dual functionality—antibacterial activity, including against resistant and infectious strains, combined with safe interaction with skin cells, which positions the developed membranes as a novel, biofunctional alternative to conventional antibiotics.

It is concluded that the PVA/SA/ZnO/BG membranes developed in this study present a promising and multifunctional approach to managing infected chronic wounds, while contributing to the reduction of antibiotic dependence. Future perspectives include the in vivo validation of these membranes in animal models, the assessment of their biodegradability, and the immunological evaluation to confirm their clinical potential further.

## Figures and Tables

**Table 1:** Composition and designation of PVA/SA with ZnO and BG nanoparticles blend solutions.

Designation	ZnO (wt.%)	BG (wt.%)
PVA/SA	0	0
PVA/SA/5ZnO	5	0
PVA/SA/10ZnO	10	0
PVA/SA/5BG	0	5
PVA/SA/10BG	0	10
PVA/SA/5ZnO/5BG	5	5
PVA/SA/5ZnO/10BG	5	10
PVA/SA/10ZnO/5BG	10	5
PVA/SA/10ZnO/10BG	10	10

**Table 2:** Thermal properties of PVA/SA and PVA/SA nanofibers with ZnO and BG NPs.

Membrane	TG Analysis		DSC Analysis			
	T_10_ (°C)	T_max_ (°C)	T_g_ (°C)	T_cc_ (°C)	T_m_ (°C)	X_c_ (%)
PVA/SA	223	255	84	nd	180	4
PVA/SA/5ZnO	229	256	84	nd	172	2
PVA/SA/10ZnO	234	255	84	nd	169	0
PVA/SA/5BG	222	268	85	120	191	7
PVA/SA/10BG	229	270	84	130	198	10
PVA/SA/5ZnO/5BG	240	270	86	123	191	9
PVA/SA/5ZnO/10BG	231	276	84	128	194	9
PVA/SA/10ZnO/5BG	232	271	86	125	191	8
PVA/SA/10ZnO/10BG	251	272	84	133	194	8

T_10_: decomposition temperature at 10% weight loss. T_max_: temperature for maximum rate of weight loss. T_g_: glass transition temperature. T_cc_: cold crystallization transition temperature. T_m_: melting transition temperature. X_c_: crystallinity percentage. Equipment error ± 2 °C. DSC: differential scanning calorimetry. TGA: thermogravimetric analysis. nd: not detected.

## Data Availability

The original contributions presented in this study are included in the article/Supplementary Material. Further inquiries can be directed to the corresponding author.

## Supplementary Materials

The following supporting information can be downloaded at https://www.mdpi.com/article/10.3390/polym17162185/s1, Figure S1: SEM images, fiber diameter histogram, and pore area distribution of PVA/SA membranes containing 5–10 wt.% of ZnO and/or BG nanoparticles; Figure S2: TGA thermograms of PVA/SA membranes containing (a) 5–10 wt.% of ZnO and/or BG, and (b) combination of different weight ratios of ZnO and BG nanoparticles; Figure S3: Differential thermogravimetric (DTG) curves of PVA/SA membranes containing (a) 5–10 wt.% of ZnO and/or BG, and (b) combination of different weight ratios of ZnO and BG nanoparticles; Figure S4: DSC second heating curves of PVA/SA membranes containing (a) 5–10 wt.% of ZnO and/or BG, and (b) combination of different weight ratios of ZnO and BG nanoparticles; Figure S5: DSC cooling curves of PVA/SA membranes containing (a) 5–10 wt.% of ZnO and/or BG, and (b) combination of different weight ratios of ZnO and BG nanoparticles; Table S1: Average diameter of fibers and pore size of PVA/SA membranes containing 5–10 wt.% of ZnO and/or BG nanoparticles; Table S2: Mechanical properties of PVA and PVA/SA nanofibers with ZnO and/or BG nanoparticles.
