# Supplementary material for: Electrospun Polyvinyl Alcohol/Sodium Alginate Nanocomposite Dressings Loaded with ZnO and Bioglass: Characterization, Antibacterial Activity, and Cytocompatibility
Source: Polymers (Basel). 2025 Aug 9;17(16):2185. doi: 10.3390/polym17162185 (PMC12388954; doi:10.3390/polym17162185)
Supplement: Supplementary file 1 [file polymers-17-02185-s001.zip › polymers-3792730-supplementary.pdf]

Electrospun Polyvinyl Alcohol/Sodium Alginate Nanocomposite Dressings Loaded with ZnO and Bioglass: Characterization, Antibacterial Activity, and Cytocompatibility

J. Andrés Ortiz*[a], Francesca Antonella Sepúlveda[b], Siomara Flores[c], Marcela Saavedra[c], Suhelen Sáez-Silva[a], Thomas Jiménez[a], Paola Murgas[d], Scarlett Troncoso[e], Camila Sanhueza[e], María T. Ulloa[e], Lorena Porte Torre[f], Manuel Ahumada [g,h], Teresa Corrales[i], Humberto Palza[b,j], Paula A. Zapata[c].

Supplementary figures and tables

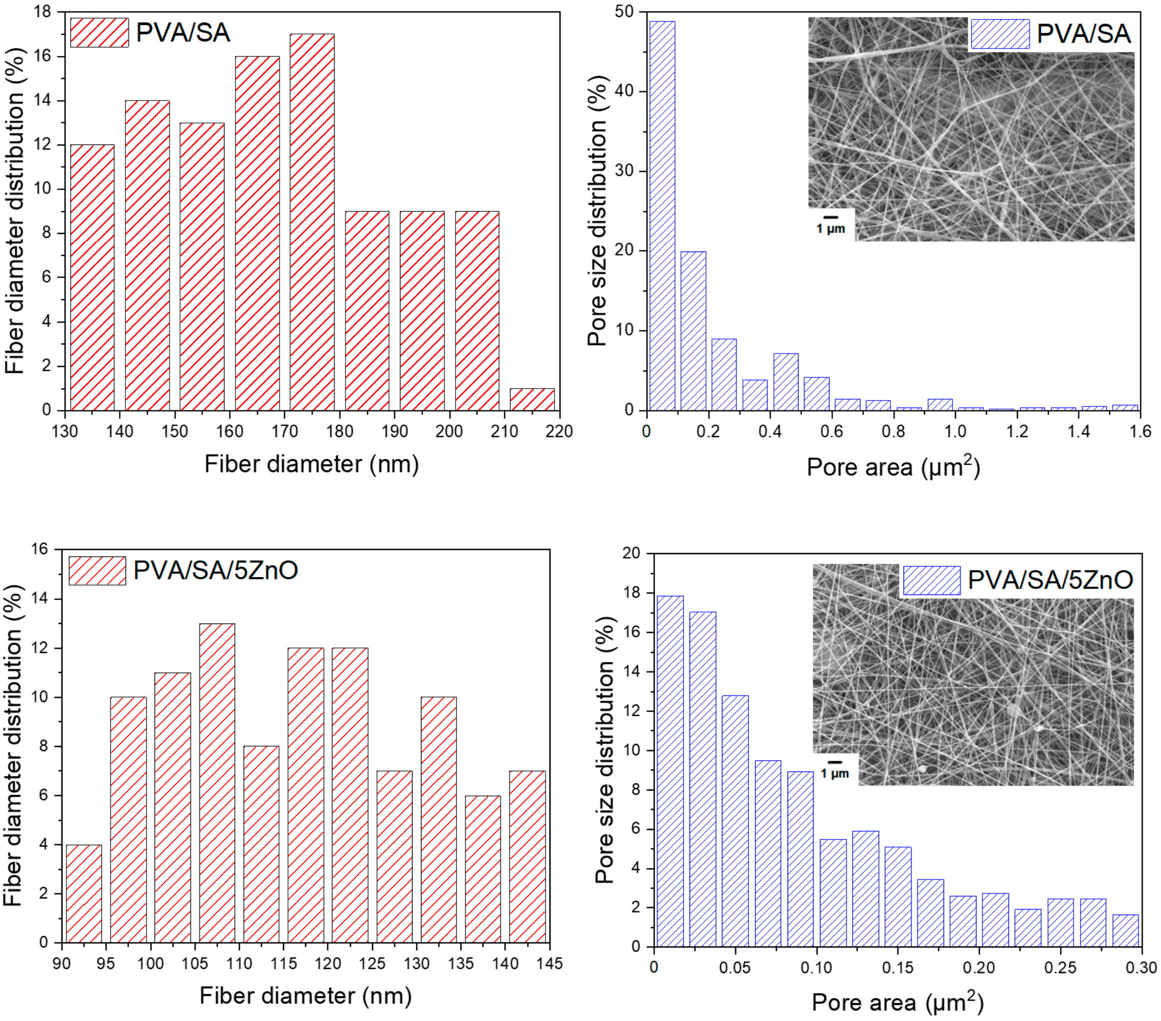

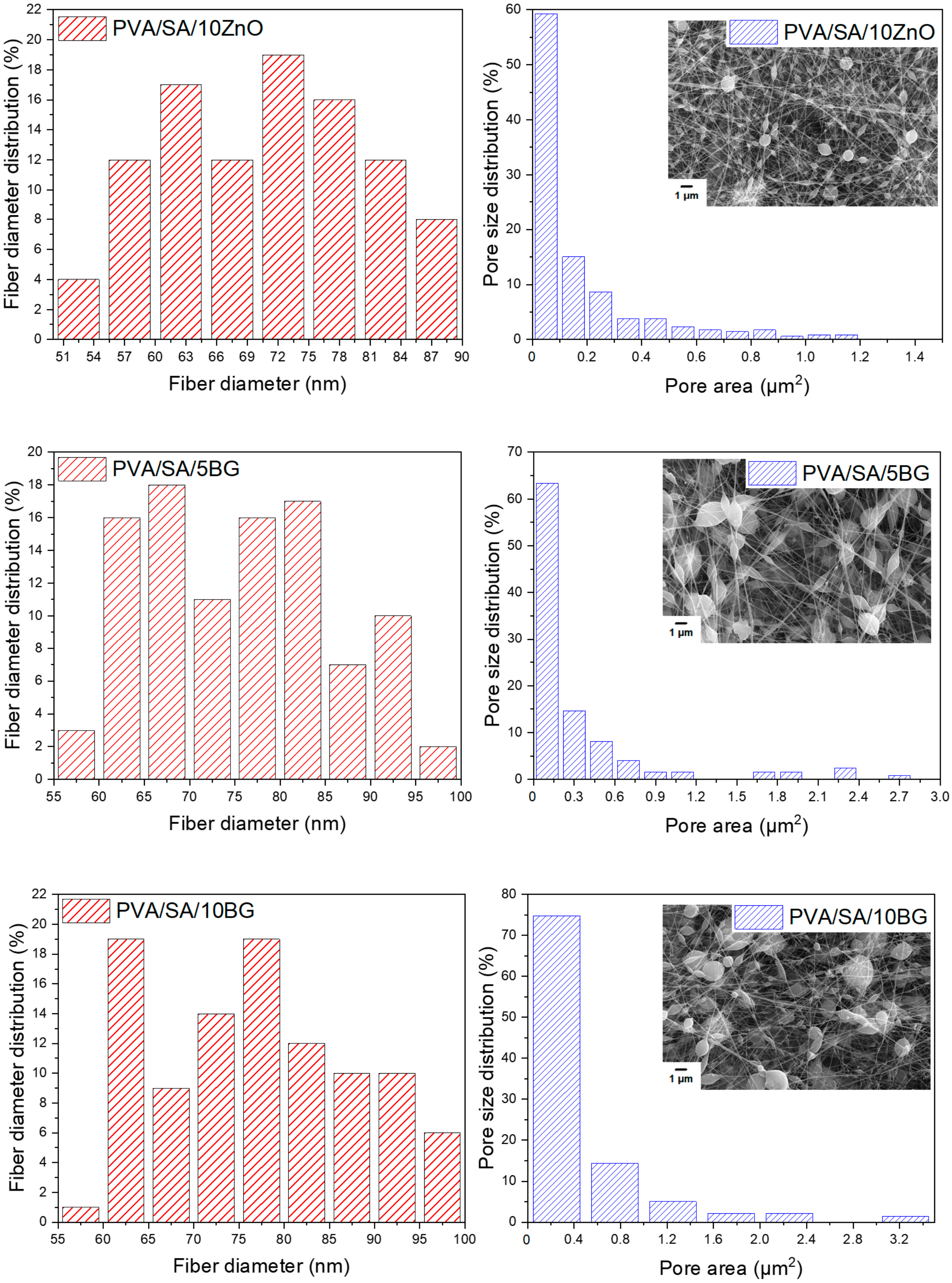
PVA/SA/10ZnO
Fiber diameter distribution (%)
Fiber diameter (nm)
PVA/SA/10ZnO
1 µm
Pore size distribution (%)
Pore area (µm²)
PVA/SA/5BG
Fiber diameter distribution (%)
Fiber diameter (nm)
PVA/SA/5BG
1 µm
Pore size distribution (%)
Pore area (µm²)
PVA/SA/10BG
Fiber diameter distribution (%)
Fiber diameter (nm)
PVA/SA/10BG
1 µm
Pore size distribution (%)
Pore area (µm²)

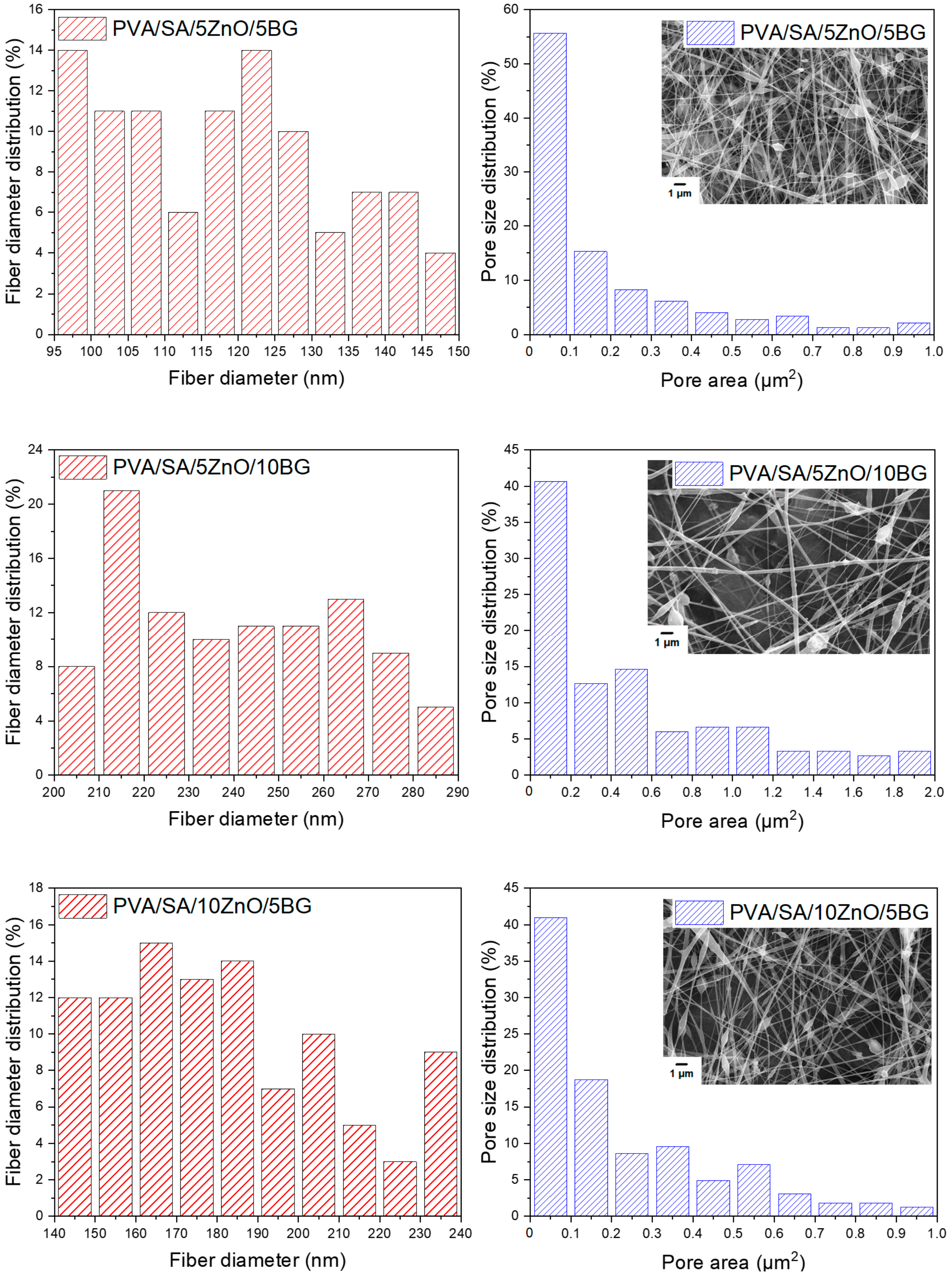
PVA/SA/5ZnO/5BG
Fiber diameter distribution (%)
Fiber diameter (nm)
PVA/SA/5ZnO/5BG
1 μm
Pore size distribution (%)
Pore area (μm^2)
PVA/SA/5ZnO/10BG
Fiber diameter distribution (%)
Fiber diameter (nm)
PVA/SA/5ZnO/10BG
1 μm
Pore size distribution (%)
Pore area (μm^2)
PVA/SA/10ZnO/5BG
Fiber diameter distribution (%)
Fiber diameter (nm)
PVA/SA/10ZnO/5BG
1 μm
Pore size distribution (%)
Pore area (μm^2)

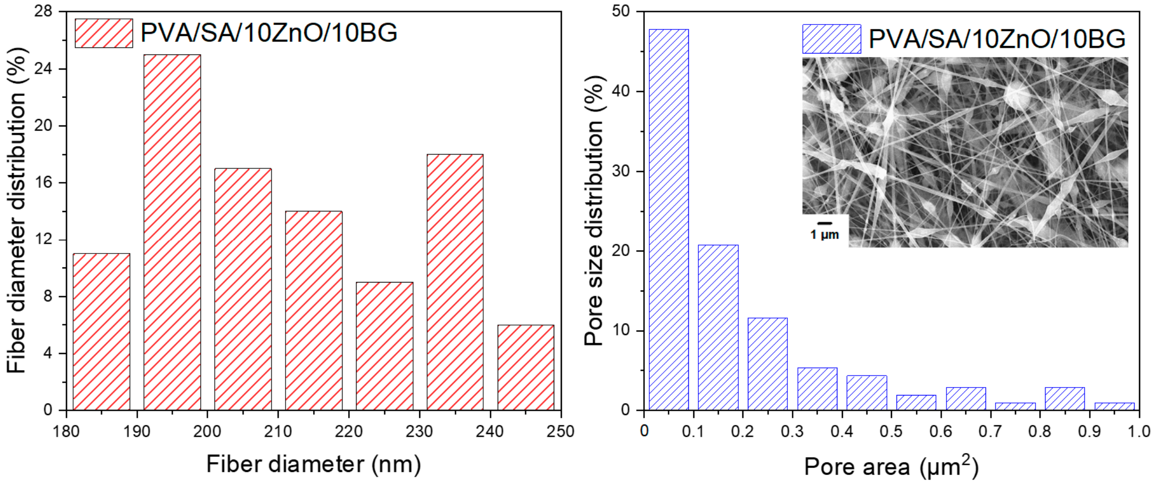

Figure S1. SEM images, fiber diameter histogram, and pore area distribution of PVA/SA membranes containing 5-10 wt% of ZnO and/or BG nanoparticles.

Table S1. Average diameter of fibers and pore size of PVA/SA membranes containing 5-10 wt% of ZnO and/or BG nanoparticles.

Membrane	Fiber Diameter (nm)	Pore area (μm^2)
PVA/SA	166 ± 22	0.06 ± 0.03
PVA/SA/5ZnO	116 ± 15	0.06 ± 0.02
PVA/SA/10ZnO	70 ± 10	0.07 ± 0.03
PVA/SA/5BG	75 ± 10	0.29 ± 0.04
PVA/SA/10BG	77 ± 11	0.23 ± 0.04
PVA/SA/5ZnO/5BG	118 ± 15	0.10 ± 0.03
PVA/SA/5ZnO/10BG	240 ± 24	0.62 ± 0.12
PVA/SA/10ZnO/5BG	182 ± 27	0.32 ± 0.11
PVA/SA/10ZnO/10BG	211 ± 18	0.24 ± 0.11

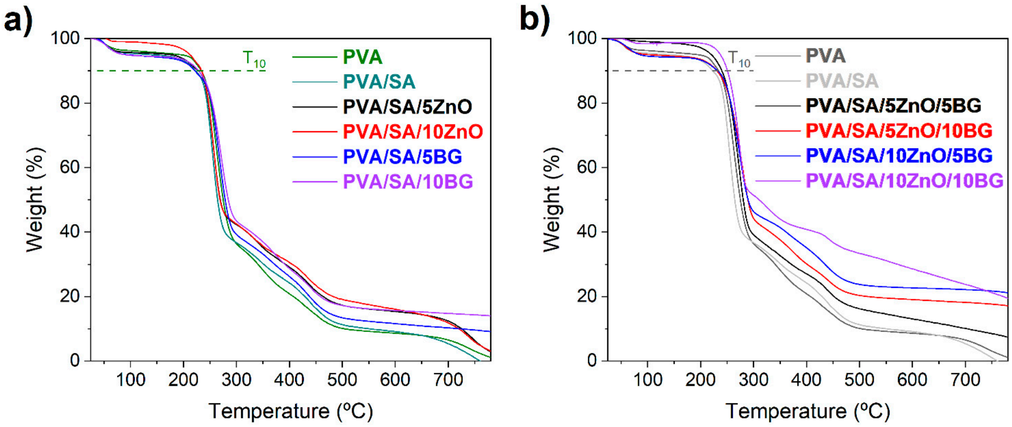

Figure S2. TGA thermograms of PVA/SA membranes containing a) 5-10 wt% of ZnO and/or BG, and b) combination of different weight ratios of ZnO and BG nanoparticles.

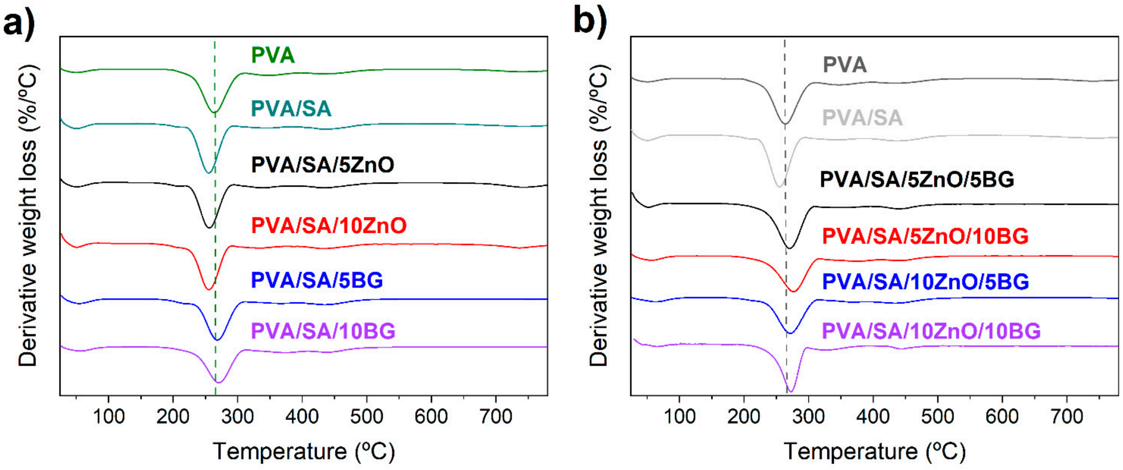

Figure S3. Differential thermogravimetric (DTG) curves of PVA/SA membranes containing a) 5-10 wt% of ZnO and/or BG, and b) combination of different weight ratios of ZnO and BG nanoparticles.

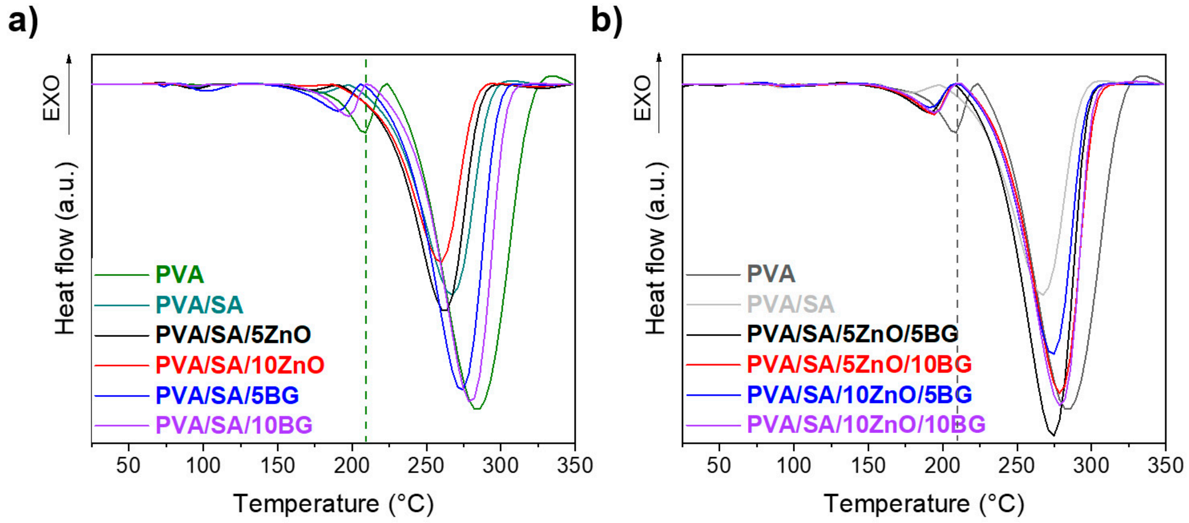

Figure S4. DSC second heating curves of PVA/SA membranes containing a) 5-10 wt% of ZnO and/or BG, and b) combination of different weight ratios of ZnO and BG nanoparticles.

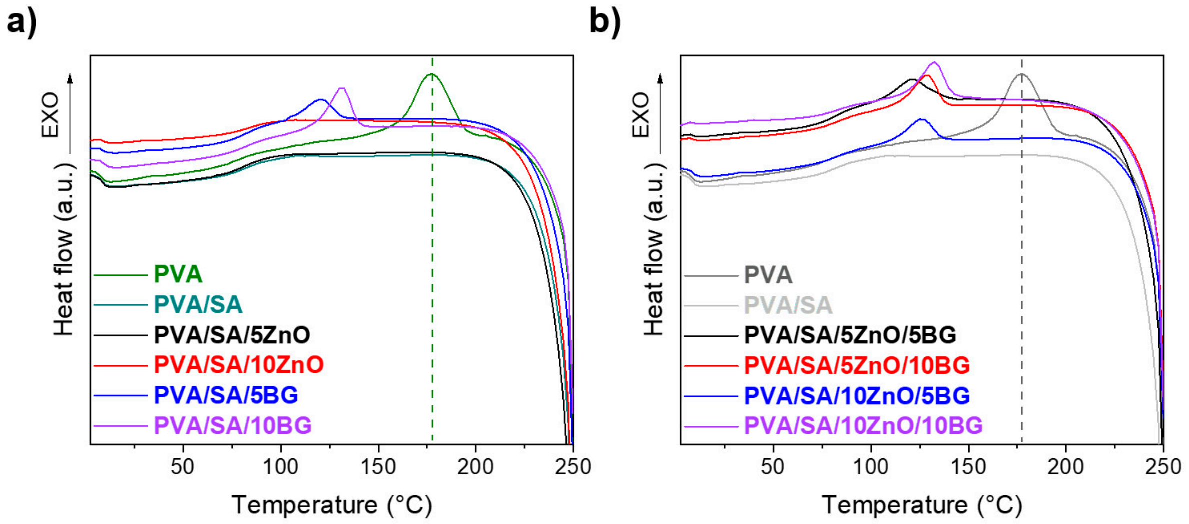

Figure S5. DSC cooling curves of PVA/SA membranes containing a) 5-10 wt% of ZnO and/or BG, and b) combination of different weight ratios of ZnO and BG nanoparticles.

Table S2. Mechanical properties of PVA and PVA/SA nanofibers with ZnO and/or BG nanoparticles.

Membrane	Young's modulus (MPa)	Tensile strength (MPa)	Elongation at break (%)
PVA	4.9 ± 0.9	0.7 ± 0.1	18.9 ± 4.3
PVA/SA	15.9 ± 2.0	1.1 ± 0.1	5.4 ± 0.6
PVA/SA/5ZnO	14.3 ± 1.9	1.2 ± 0.2	3.8 ± 0.3
PVA/SA/10ZnO	5.6 ± 1.3	0.5 ± 0.1	4.5 ± 1.0
PVA/SA/5BG	3.6 ± 1.9	0.2 ± 0.1	4.2 ±0.9
PVA/SA/10BG	31.8 ± 6.3	0.4 ± 0.3	2.5 ± 1.5
PVA/SA/5ZnO/5BG	20.2 ± 4.4	0.7 ± 0.2	4.1 ± 1.8
PVA/SA/5ZnO/10BG	21.1 ± 2.3	0.5 ± 0.1	2.7 ± 1.3
PVA/SA/10ZnO/5BG	14.8 ± 1.9	0.6 ± 0.2	3.9 ± 0.9
PVA/SA/10ZnO/10BG	7.9 ± 1.6	0.2 ± 0.1	2.5 ± 0.8
